# Development of Peptide Biopharmaceuticals in Russia

**DOI:** 10.3390/pharmaceutics14040716

**Published:** 2022-03-27

**Authors:** Vladislav I. Deigin, Elena A. Poluektova, Allan G. Beniashvili, Sergey A. Kozin, Yuri M. Poluektov

**Affiliations:** 1 Shemyakin-Ovchinnikov Institute of Bioorganic Chemistry, Russian Academy of Sciences, 142290 Moscow, Russia; vdeigin@gmail.com; 2 Department of Propaedeutics of Internal Diseases, Gastroenterology and Hepatology, I.M. Sechenov First Moscow State Medical University (Sechenov University), 119991 Moscow, Russia; polouektova@rambler.ru; 3 Mental Health Research Center, Federal State Budgetary Scientific Institution, Ministry of Health of the Russian Federation, 115522 Moscow, Russia; beniashvilia@yandex.ru; 4 Engelhardt Institute of Molecular Biology, Russian Academy of Sciences, 119334 Moscow, Russia; kozinsa@gmail.com

**Keywords:** peptide synthesis, peptide manufacturing, peptide drug, peptidomimetics, cyclopeptide

## Abstract

Peptides are low-molecular-weight substances that participate in numerous important physiological functions, such as human growth and development, stress, regulation of the emotional state, sexual behavior, and immune responses. Their mechanisms of action are based on receptor–ligand interactions, which result in highly selective effects. These properties and low toxicity enable them to be considered potent drugs. Peptide preparations became possible at the beginning of the 20th century after a method was developed for selectively synthesizing peptides; however, after synthesis of the first peptide drugs, several issues related to increasing the stability, bioavailability, half-life, and ability to move across cell membranes remain unresolved. Here, we briefly review the history of peptide production and development in the biochemical industry and outline potential areas of peptide biopharmaceutical applications and modern approaches for creating pharmaceuticals based on synthetic peptides and their analogs. We also focus on original peptide drugs and the approaches used for their development by the Russian Federation.

## 1. Introduction

Studies of the chemical structures and biological properties of peptides and proteins became possible at the beginning of the 20th century, when Fischer [1] and colleagues developed a method for selectively synthesizing peptides comprising several amino acids. The use of peptides as drugs has evolved and continued to develop, and the molecular characteristics and structures of receptors for many important endogenous peptides have been identified [2,3]. In addition, the sequences of new peptide molecules have been determined, which can serve as a basis for identifying new peptide biomarkers of various diseases [4].

Currently, ~70 peptide-based drugs are registered in the international pharmaceutical industry [5] (Supplementary Materials Table S1), and 14 products are registered in the Russian Federation (Table 1). The versatility of peptide functions in the human body has made it possible to create drugs (based on natural molecules and their modified analogs) that are approved for use in a wide range of indications from oncology to dentistry. Peptides that affect the functions of the central nervous system, including neurotransmitters and endogenous opioid peptides, have attracted the most interest.

## 2. Nomenclature, Classification, and the Roles of Endogenous Peptides

Peptides are mainly categorized in three different ways: (1) According to how many amino acids make up the chain (molecules comprising ≥50 amino acids linked together in chains by peptide bonds are called proteins, and shorter molecules are called peptides. Peptides are also subdivided into polypeptides (20–50 amino acids), oligopeptides (10–20 amino acids), and short or mini-peptides (2–0 amino acids)); (2) according to peptide source (plant, animal, or external (e.g., a marine source)); (3) or according to their functions in the human body [6,7,8,9]. For structure–activity studies, peptides are often classified by their structures as linear peptides, cyclopeptides, multifunctional peptides, cell-penetrating peptides, or peptide–drug conjugates [10]. Additionally, classification is often made according to peptide physiological effects [11]. These divisions are somewhat arbitrary, and with the development of improved technological methods for generating proteins and peptides, the differences have gradually diminished. 

Peptides represent a prime example of the functional diversity of a protein encoded by a single gene. For example, the insulin hormone processed in the body from proinsulin, as following cleavage of C-peptide [12], the proopiomelanocortin protein [13], forms the basis for various neuropeptides that interact with the opioid receptors µ, δ, and κ [14]. Many peptide hormones (i.e., vasopressin, prolactin, oxytocin, adrenocorticotropic hormone (ACTH), bradykinin, melanocyte-stimulating hormone, oxytocin, and glucagon) have synthesized as prohormones [15]. Neuropeptides comprise the most important class of endogenous peptides [16], are synthesized in the central and peripheral nervous systems, and help regulate most physiological processes. 

In addition, endogenous peptides participate in regulating emotional states, sexual behavior, sleep, wakefulness [17], and immune responses [10,18]. Certain peptides promote the elimination of radionuclides and heavy metal salts from the body [8]. Many hormones (i.e., vasopressin, prolactin, oxytocin, ACTH, bradykinin, melanocyte-stimulating hormone, oxytocin, and glucagon) have a peptide nature [15].

## 3. Therapeutic Applications of Peptides

**Figure 1:**
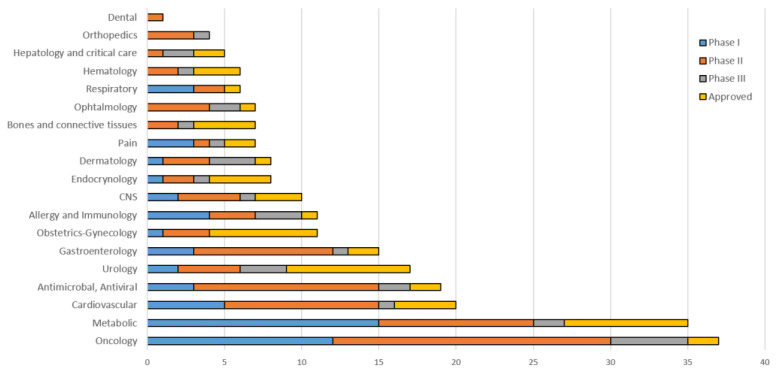
Peptides approved and in active development by therapeutic area.

The strategies for using peptide drugs have shifted from hormonal therapy and diagnosing oncologic diseases towards treatments of a wide range of diseases, such as diabetes mellitus, osteoporosis, cardiovascular diseases, functional gastrointestinal diseases, and multiple sclerosis [10,11,28]. The current status of peptide drug development in different areas of the medicine is listed in Figure 1 (THPdb: A database of FDA approved therapeutic peptides and proteins).

In particular, growing interest exists in using natriuretic peptides as a novel non-invasive biomarker of heart failure and in developing stable forms of neuropeptides that would enable their use as potent medicines [19]. Neuropeptides are considered biomarkers or drugs for diseases associated with impaired regulation of energy balance, food–behavioral reactions, and mental disorders. Enkephalins and their derivatives are considered candidates for treating chronic pain, maladjustment, and pathological stress responses [20]. Neuropeptide Y is a prospective molecule for treating various diseases of the central nervous system, cardiovascular and endocrine systems, and respiratory and gastrointestinal tracts [21].

Pharmacological analogs of somatostatin are increasingly being used to treat cancer, acute pancreatitis, and acromegaly [22]. Diagnostic and treatment approaches that target cholecystokinin, such as receptor scintigraphy and radiopharmaceuticals, have been used in tumor imaging and/or therapy in vitro, in vivo, and in clinical studies [23]. Pharmacological analogs of cholecystokinin have been studied for applications in oncology, addiction, and eating disorders. Additionally, the presence of intestinal peptide receptors in immune cells and vagus nerve endings opens up new targets for pharmacological approaches for addressing aging and mental disorders [24]. Moreover, studies on treating neurodegenerative diseases using peptides have also been conducted [25]. Furthermore, bacteriocins are considered promising agents for treating antibiotic resistant strains of pathogenic bacteria, human defensins are being studied as antibacterial drugs [26,27], and many peptides are being evaluated as potential therapeutic agents in oncology research [28].

## 4. Problems Associated with Manufacturing Synthetic Peptides

Synthetic peptide drugs entered the global pharmaceutical market in the 1960s, and modern technologies enable the isolation and assessment of animal and plant peptides, as well as antimicrobial peptides from amphibians and microorganisms, as candidate therapeutic agents [29]. Nevertheless, most peptide preparations (~85%) are obtained by chemical synthesis, and only 15% are obtained by recombinant methods. Furthermore, chemical-synthesis technologies also provide opportunities to modify peptides using non-natural amino acids and introduce pseudopeptide bonds and other modifications not available using recombinant techniques [30].

Of the small-molecule chemicals prevailing in the global pharmaceutical market (85%), peptides represent only a small portion (2%) of the world drug market. However, the market for peptide- and protein-based drugs is growing approximately twice as rapidly as the rest of the drug market, indicating that peptides may soon occupy a more significant niche [11,31]. During the initial stages of peptide pharmaceutical development, a scarcity of registered peptide drugs was caused by several objective factors [32], including high production costs, low bioavailability when taken orally, insufficient stability, an insufficient ability to traverse cell membranes, and short half-lives. However, the advantages of peptide molecules include a high selectivity and affinity for the corresponding receptor, low toxicity and immunogenicity, as well as multiple biological targets in the body and a low likelihood of cross-interactions with other drugs.

In the previous 15 years, new synthesis strategies have emerged to enable changes in the pharmacokinetic properties and specificity of peptide molecules by modifying amino acids or the peptide chain, incorporating non-natural amino acids, conjugating peptides with carriers that increase the half-life, and/or improving peptide solubility. In addition, new targeted drug-delivery strategies have enhanced the stability and other physical and chemical properties of potential peptide drugs [11].

## 5. The Main Strategies for Developing the Next Generation of Peptide Drugs

The approaches used to increase peptide stability are continuously being improved, leading to new kinds of structural modifications [33,34]. One apparent solution for stabilizing the hydrolytic lability of drugs containing natural peptides is to incorporate modified analogs of natural peptides previously registered as parenteral drugs. Analog modifications are based on introducing substitutions in various parts of the original molecule in order to stabilize and sometimes change its structure, spectrum, and even direction of action [35].

An essential requirement for improving peptide structure is an ability to minimize the possible toxicity of the obtained analogs. Currently, many laboratories [36] are developing peptide-modification strategies to increase the binding affinity to receptors or active centers of enzymes, as well as their absorption, distribution, metabolism, and excretion profile (known as the “ADME” profile) [37].

Novel synthetic strategies allow for modulating pharmacokinetic properties and target specificity through amino acid or backbone modification by incorporating non-natural amino acids and conjugating moieties that extend half-life or improve solubility. Substituting natural amino acids is one strategy used to prevent hydrolysis, where modifications are introduced at sites that undergo hydrolysis, followed by replacing the original amino acid [3]. The substituents can be d-amino acids, β-amino acids, dehydroamino acids, and various olefin derivatives. Such modifications improve the stability and increase the half-life of the peptide molecules in plasma [11,38]. Various critical issues associated with therapeutic peptide delivery have drawn increasing attention to the development of new formulations for alternative routes of administration, such as oral, nasal, buccal, pulmonary, transdermal, rectal, and ocular [39]. Penetration of drugs through oral mucosa into the systemic circulation is a major hindrance in their absorption, as the oral route easily degrades a hydrophilic, large-molecular-weight drug (e.g., proteins and peptides), resulting in their decreased availability in systemic circulation [40].

Examples of modifications include the introduction of proline and hydroxyproline (both resistant to protease degradation) into cleavage sites to replace easily hydrolyzed amino acids in order to improve in vivo drug stability [41,42]. In addition, N-methylation or the introduction of N-methyl-amino acids has also been used to increase peptide stability, reduce possible hydrogen bonding, and improve permeability [43]. Moreover, the simultaneous inclusion of d-amino acids and N-methylation at amide bonds can significantly increase metabolic stability, thereby creating additional steric hindrance. Furthermore, many structural modifications, including N-alkylation, can increase the biological and metabolic stability of peptides [44,45].

Proteolytic enzymes in the blood, plasma, liver, or kidney include exopeptidases, aminopeptidases, and carboxypeptidases, which hydrolyze peptide sequences from N- and C-termini. Therefore, N-acylation and C-amidation can potentially increase the resistance of modified peptides to proteolysis [46]. Linear cyclization is a generally accepted method of increasing protein rigidity, with this process resulting in the formation of intramolecular hydrogen bonds and decreasing intermolecular hydration. Head-to-tail peptide cyclization offers the advantage of strengthening the peptide chain, stabilizing the conformation, and inhibiting cleavage by endopeptidases. Therefore, cyclization might represent the simplest method to prolong the half-life of a peptide in vivo, as it often increases the biological activity of a peptide [35]. Moreover, introducing N-terminal d-amino acids can suppress degradation by exopeptidases, similar to reducing C-terminal carboxyl groups into a corresponding alcohol moiety [47].

The chemical “stapling” of amino acid side chains onto a peptide chain can be achieved via the insertion of residues into a peptide chain through hydrocarbon “inserts” or by forming lactam bridges to stabilize peptide helicity and increase their stability and intracellular permeability. The so-called “stapled-peptides” method is gaining popularity [48,49]. Another modern approach to increasing peptide stability and creating a more durable compound is to conjugate peptides with macromolecules. Various polymers have been applied for these purposes, including polyethylene glycol (PEG) [50] and polyvinylpyrrolidone, as well as the use of protein carriers, such as albumin. PEGylating peptides can effectively reduce their potential immunogenicity, maintain their biological activity, and slow down enzymatic hydrolysis [51]. In addition, some fatty acids are used to stabilize peptides and protect them against proteolysis. Peptide molecules are encapsulated into liposomes, nano/microparticles, or micelles with a higher molecular weight [52] to increase the half-lives and bioavailabilities of peptide drugs [53].

Conjugating peptides with lipids confer lipopeptide derivatives with new structural and biological properties that result in compounds with improved potency and selectivity. Lipidation of peptides leads to the formation of amphiphilic peptide conjugates with increased bioavailabilities and increased capability to cross cell membranes [54]. Recently, a new concept for creating full-length enantiomeric d-peptides, which involves replacing all l-amino acids with the corresponding d-amino acids, has become widespread, with such peptides (d-peptides) showing significantly improved stabilities and half-lives [55,56].

**Figure 2:**
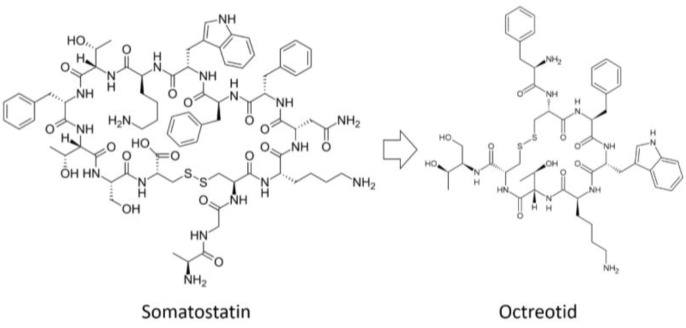
Analog of somatostatin with d-amino acids.

One of the first natural peptides to be successfully modified was the hormone vasopressin, which contains l-Arg and has a half-life in humans of 10 to 35 min [57]. Vasopressin analogs containing d-Arg instead of l-Arg are called desmopressin and have a half-life of ~4 h [58]. An analog of somatostatin (the drug octreotide, which is used to treat gastrointestinal tumors) has a shorter sequence than somatostatin (8 amino acids instead of 14) and l-amino acid substitutions for the corresponding d-amino acids (Figure 2) [59].

**Figure 3:**
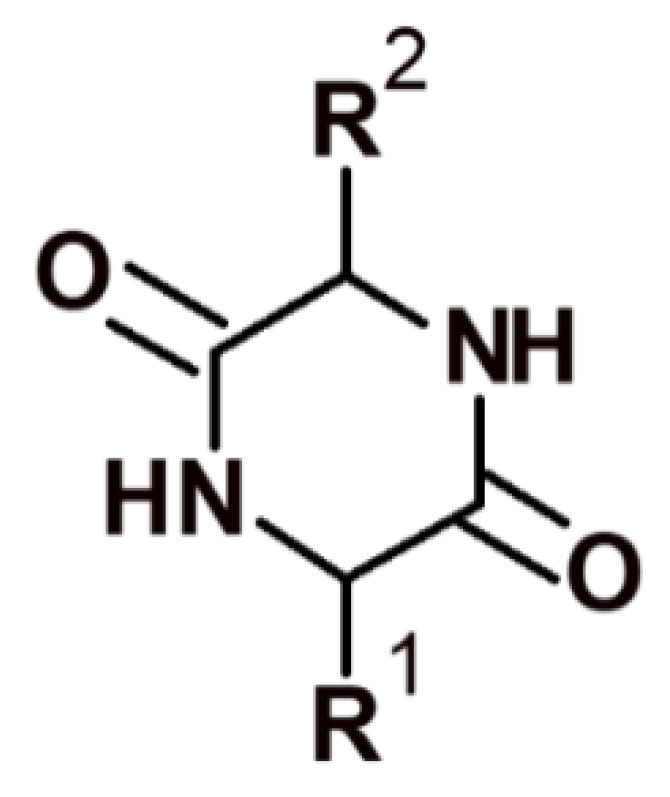
Structure of 2,5-diketopiperazine.

The minimal cyclic structures of peptide compounds are 2,5-diketopiperazines (DKPs), which are cyclodipeptides obtained by condensing two α-amino acids (Figure 3).

Numerous different structures can be generated based on DKP in order to search for new lead compounds [60]. DKP derivatives are often found in nature both in the form of simple unsubstituted 2,5-DKP structures and more complex molecular structures in natural products, fungi, bacteria, plants, and mammals. For example, many antibiotics are DKP derivatives [61]. Drugs have been developed with structures ranging from simple cyclic dipeptides, such as derivatives of cycloserine dimers [62] or kairomycin B [63], to complex conjugated polynuclear systems, such as bicyclomycin [64].

**Figure 4:**
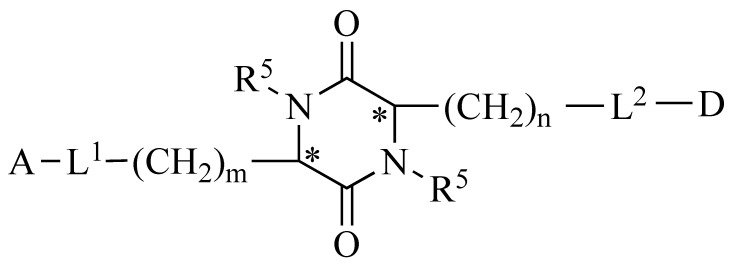
The general formula of a platform for synthesizing libraries of peptidomimetics based on substituted 2,5-DKP. A and D represent biologically active pharmacophores or fragments of peptide compounds; L^1^ and L^2^ are biodegradable linkers; m and n are the number of CH_2_ groups (ranging from 0 to 4); and R^5^ represents possible derivatives of the pharmacophore attached at the nitrogen atoms. * Denotes regions where the S or R optical orientation is possible at the carbon atoms at positions 3 and 6.

2,5-DKP is resistant to proteolysis and an attractive target for structural and functional studies aimed at searching for new potential drugs. These conformationally limited chiral centroids have six positions available for structural modification by various functional groups with specific stereochemistry. The 2,5-DKP structure enables alterations at all six positions and stereochemical isomerization at all four positions of the optical centers. In addition, 2,5-DKP has a rigid framework that can mimic the preferred conformation by limiting the mobility of amino acids embedded in its structure. The 2,5-DKP structure comprises trifunctional amino acids containing various functional groups, which can be used to identify target positions with which this molecule interacts and to serve as linkers for attaching multiple functional groups (pharmacophores) (Figure 4).

We previously developed an original platform for modifying peptides by synthesizing peptidomimetics based on substituted 2,5-DKP [65,66,67]. Pharmacophores should be able to easily undergo metabolic transformations, such as ester bond formation with the centroid and easy hydrolysis in the body. 2,5-DKPs are relatively easy to synthesize and can accommodate a wide variety of substituents (i.e., various amino acids used as building blocks). The large set of substituents makes it possible to widely vary the physicochemical characteristics of the molecule, including its structure, size, shape, lipophilicity, dipole moment, electrostatic charge, and functional groups. This flexibility enables in silico modeling of analogs for directed library design [68].

When looking for new lead compounds, it is critical to not introduce changes in the centroid or substitutions in the attached groups that can lead to toxicity. One reason explaining the different physiological activities of drug stereoisomers is the differences in their penetration into an organism, which may be due to the structural features of 2,5-DKP, the properties of biological membranes (which are produced from optically active, asymmetric material), and the presence of transport systems that transport metabolites across membranes [69]. In one approach that utilizes 2,5-DKPs [70] (both short peptide analogs and versions containing “inserts” at different positions in the chain), the DKP moiety is positioned at the N- or C-terminal end of the molecule or within the peptide. The use of this approach has become widespread and can increase the hydrolytic stability and possibility of oral administration [71]. Furthermore, some derivatives of branched DKPs can exhibit hemostimulatory [65] and immunosuppressive properties [66], with one study demonstrating an acquisition of several new drugs based on 2,5-DKPs [68].

## 6. Primary Strategies for Developing the Next Generation of Peptide Drugs in Russia

**Figure 5:**
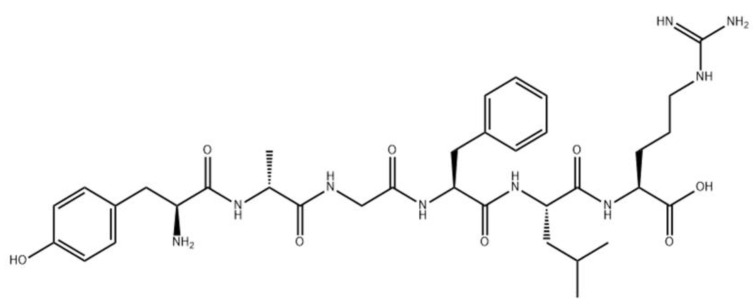
Chemical structure of dalargin.

At the end of the 1960s, several laboratories that focused on the chemistry of natural compounds were started in the former Union of Soviet Socialist Republics (USSR), with these including several laboratories engaged in studying peptides, developing novel active peptide analogs, and improving technological methods for creating peptide-based drugs. Thus, research on synthesizing peptides and their derivatives, the structures of which were already established, was actively conducted, resulting in the production of oxytocin, vasopressin, ACTH, somatostatin [72,73], as well as depsipeptides, inofors, and other peptide compounds. The pioneers in this field were scientists from Moscow (Institute of Chemistry of Natural Compounds of the USSR Academy of Sciences; now the M. M. Shemyakin and Yu. A. Ovchinnikov Institute of Bioorganic Chemistry Russian Academy of Sciences (RAS), Moscow Lomonosov State University, Institute of Molecular Genetics of the USSR Academy of Sciences, Institute of Pharmacology of the USSR Academy of Medical Sciences, and the Institute of Highly Pure Bioproducts). The first original peptide drugs developed in the USSR were dalargin [74] (Figure 5) and thymogen [75,76], with dalargin representing the world’s first synthetic neuropeptide drug created as an analog of leucine-enkephalin. 

Thymogen is a natural immunocorrector. Initially, the peptide was isolated from a thymus extract and subsequently obtained synthetically. Industrial technology for manufacturing peptide drugs was subsequently developed, and peptide production was launched at the number of pharmaceutical plants. At the same time, chemists investigated the synthesis and creation of new original drugs in several other Russian laboratories and institutes [55]. 

Comparing the achievements of Russian developers during this timeframe with those of developers from other countries suggests that of the ~5000 chemical preparations produced, ~15 were Russian-made (~0.1%), and of 70 peptide preparations, 14 were Russian, accounting for almost 20% of worldwide development (Table 1).

## 7. Ongoing Clinical Trials for Peptide Drug Applications in Russia

Currently, most research associated with peptide drug applications is aimed at Alzheimer’s disease, inflammation, and cerebral ischemia.

### 7.1. Drugs Targeting AD

Proteinopathies, the most famous of which are involved in AD pathogenesis, are characterized by an abnormal structural rearrangement and subsequent aggregation of certain protein molecules [77], and are of particular interest in terms of using peptides as disease-modifying drugs. Studies suggest that short peptides or peptidomimetics that specifically bind to protein molecules prone to spontaneous aggregation (amyloid beta (Aβ) in the case of AD) can stop this pathological process [78]. To treat AD, the Aβ fragments 13-HHQK-16 [79] and 16-KLVFF-20 [80] have been extensively evaluated as drug targets, resulting in the generation of the peptidomimetic tramiprosate [78] and so-called β-sheet-breaker peptides [81]; however, these did not show therapeutic effects in clinical trials.

Since 2010, the Laboratory of Protein Conformational Polymorphism in Health and Disease (Engelhardt Institute of Molecular Biology, RAS, under the guidance of Professor A. A. Makarov) has performed in silico, in vitro, and in vivo studies, focusing on elucidating the role of Aβ structural polymorphisms caused by modifying amino acids in the metal-binding domain (amino acids 1–16) in the evolution of the key molecular process of AD; namely, the conformational transformation of Aβ from a physiologically normal monomeric state to insoluble aggregates saturated with zinc, iron, and copper ions. Based on the established molecular mechanisms of zinc-dependent interactions of Aβ with biomolecules important for various classes [82,83,84,85,86,87,88,89], it was determined that the 11-EVHH-14 fragment is a molecular determinant of Aβ [90]. Furthermore, the 11-EVHH-14 region has a relatively rigid backbone conformation in soluble Aβ monomers [91,92] and zinc-bound dimers [82]. This site corresponds to β-strand β2 from the N-terminal arch of the Aβ amyloid fibrils purified from brain tissues from patients with AD and is solvent-exposed and accessible for interactions with external molecules [93].

It is known that Aβ interacts with nicotinic acetylcholine receptors (nAChRs), and it was recently shown that it is the 11-EVHH-14 region of Aβ that is critical for interactions with α4β2- and α7-containing nAChRs [94]. Thus, molecular agents that bind to the 11-EVHH-14 region of Aβ can inhibit such interactions and, therefore, act as a potential therapeutic for the treatment of cholinergic dysfunction in AD. In Makarov’s laboratory, it was found by using bioinformatics and molecular modeling that the a4 subunit of α4β2-nAChR contains a tetrapeptide site 35-HAEE-38, which is ion-complementary to the 11-EVHH-14 region of Aβ [95]. Surface plasmon resonance experiments confirmed that the synthetic peptide [Acetyl]-His-Ala-Glu-Glu-[Amide] (Ac-HAEE-NH2), in which the N- and C-termini are protected by acetyl and amide groups, respectively, specifically binds to 11-EVHH-14 site within the Aβ metal-binding domain. Then, it was shown that amyloid aggregates formed upon contact with α4β2 nAChR in model oocytes and blocked the normal function of receptors and that these effects were prevented by exposure to exogenous Ac-HAEE-NH2 molecules [25]. Intravenous injections of the Ac-HAEE-NH2 peptide dramatically slowed cerebral amyloidogenesis in B6C3-Tg (APPswe, PSEN1-dE9) 85Dbo/j mice, which are used as an animal model of AD [96]. The application of therapeutic peptides for treating AD has been widely discussed [97]; however, the ability of such peptides to penetrate the blood–brain barrier (BBB) is limited [98]. Using pharmacokinetics and molecular modeling, Makarov et al. [99] showed that Ac-HAEE-NH2 peptides can pass through the BBB. Notably, the role of low-density-lipoprotein receptor-related protein 1 in receptor-mediated transcytosis of Ac-HAEE-NH2 has been strongly proposed.

The application of therapeutic peptides for treating AD has been widely discussed [97]; however, the ability of such peptides to penetrate the blood–brain barrier (BBB) is limited [98]. Using pharmacokinetics and molecular modeling, Makarov et al. [99] showed that HAEE peptides can pass through the BBB. Notably, the role of low-density-lipoprotein receptor-related protein 1 in receptor-mediated transcytosis of HAEE has been strongly proposed.

### 7.2. Inflammation

Inflammation results from a cascade of reactions caused by components of tissue fluid, lymph, plasma, leukocytes, platelets, endothelium, and connective tissue cells. Several cell types synthesize alarmins in response to factors that cause inflammation. Alarmins transmit information that resembles signals from endogenous sources of damage-associated molecular pattern molecules (DAMPs). Cells entering apoptosis release DAMPs that initiate a non-infectious inflammatory response, and DAMPs entering the extracellular space activate immune cells to restore homeostasis when it has been disturbed. Currently, 13 alarmins have been discovered, but many molecules produced by mono- and polynuclear phagocytes, eosinophils, mast cells, endothelial cells, and platelets have alarmin-like properties. One of the most important alarmins is the non-histone, chromosomal cytokine protein known as high-mobility group box chromosomal protein 1 (HMGB1), which was first isolated in 1999 from calf thymus tissue. The HMGB1 protein mainly localizes to the cell nucleus, where it functions as a DNA chaperone. HMGB1 is one of the most studied DAMPs and comprises 215 amino acid residues that map to three domains: two homologous DNA-binding HMGB domains (the A- and B-boxes) and a negatively-charged C-terminal region that is 30 amino acids long and consists of Asp and Glu residues. The C-terminal fragment of alarmin 1 interacts with DNA, and the B-box activates the secretion of proinflammatory cytokines by macrophages, whereas the A-box inhibits the proinflammatory reaction induced by the B-box. In the extracellular space, HMGB1 acts as a cytokine by participating in the transmission of signals related to cell division, migration, the initiation of inflammation, and the immune response [100].

In 2020, the Research Center for Biomedical Technologies of the Federal Biomedical Agency of Russia studied the peptide agonist leutragine (also known as DOR), which can prevent the release of HMGB1 into the lungs of mice induced via lipopolysaccharide (LPS) inhalation. This effect was mediated by opioid receptors, increased regulation of sirtuin 1 expression, and decreased regulation of hyperacetylation in critical lysine residues in the nuclear localization signal NLS1 and NLS2 domains of HMGB1. HMGB1 hyperacetylation is an essential prerequisite for its active release from cells into the extracellular environment. Leutragine reportedly inhibits LPS-induced HMGB1 hyperacetylation in both the NLS1 and NLS2 domains, with the greatest effect observed for HMGB1 released in bronchoalveolar lavage fluid. Thus, leutragine appears to reduce HMGB1 release into the lungs of LPS-induced mice by preventing HMGB1 hyperacetylation. These results suggest that leutragine is a promising therapeutic agent for treating pneumonia associated with the release of HMGB1 [101].

### 7.3. Cerebral Ischemia

Cerebral stroke is currently an urgent medical and social problem due to its high frequency and high rates of morbidity and mortality. In Russia, vascular brain diseases have replaced cardiovascular diseases as the most common cause of death, and ischemic brain lesions are dominant in the field of cerebrovascular pathology, accounting for up to 80% of all vascular diseases. According to the National Stroke Association in Russia, >450,000 people experience cerebral strokes annually (every 1.5 min, someone in Russia develops this disease for the first time). Up to 200,000 of these cases are fatal, and up to 80% of the surviving patients remain disabled with varying severity. Developing approaches to delay brain tissue damage during or following cerebral ischemia necessitates the use of a special kind of pathogenetic therapy (i.e., neuroprotection), which can be initiated during the prehospital stage, even considering the possible hemorrhagic nature of stroke. 

A neuropeptide with the amino acid sequence Met–Glu–His–Phe–Pro–Gly–Pro (known as “Semax”) was synthesized under the guidance of two academicians of the RAS (I. P. Ashmarin and N. F. Myasoedov) by scientists at the Institute of Molecular Genetics of the RAS, and deserves special attention. The acetylated form of the heptapeptide Semax is an analog of the ACTH (4–10) fragment. The neuroprotective effects of Semax have been established in vitro and in animal models of cerebral ischemia, as well as under clinical conditions.

The Semax peptide is used to treat ischemic stroke due to its nootropic, neuroprotective, and immunomodulatory effects. Analyzing the transcriptome with an ischemic model of transient middle cerebral artery occlusion (tMCAO) revealed increased mRNA-expression levels of many proinflammatory genes, and that Semax suppressed their induction [102]. A study was subsequently conducted showing that the protective effect of Semax in a stroke model may have been due to its anti-inflammatory effects. This study revealed the compensatory effects of the Semax peptide on inflammation- and neurotransmitter-related genetic reactions after tMCAO, which may explain the neuroprotective effect of Semax during ischemia-reperfusion and suggest an important feature of Semax as the ability to promote the normalization of ischemia-disrupted mRNA-expression patterns [103].

An important pharmacodynamic property of Semax is its low toxicity and safety. Moreover, Semax does not have a debilitating effect on the central nervous system; negatively affect parameters of the cardiovascular and other important systems of the body; affect the metabolism of other drugs; reduce resistance to mental and physical stress; or cause drug dependence, addiction, and withdrawal syndrome.

## 8. Conclusions

The interest in peptide drugs in Russia has been driven by the need for imported substitutions, the development of competitive analogs of foreign-produced drugs, and the rapid increase in the number of studies on the pathogenesis of a wide range of diseases, including cardiovascular, neurological, and oncological diseases. The formation of a solid theoretical basis enables the selection and prediction of targets for developing new peptide drugs. Considering the possibility of increased funding for the peptide market, the accumulation of fundamental knowledge and the experience of previous developments promote the rapid development of this industry. 

The lack of international recognition of developments in this field by Russian scientists is due to a significant lag in the implementation of so-called “good industry practices”. The active period of the formation of international practices, including good laboratory practices and good clinical practices, in most countries of Western Europe occurred during the 1980s and 1990s. Since then, a new generation of scientists with the necessary competencies has emerged in these territories. During this period, the pace of scientific development lagged that of other countries, leading to the erosion of the educational system, a lack of continuity, and a shortage of specialists with the necessary expertise in the field of modern international practices. The harmonization between the regulatory field and industrial-practice standards could help eliminate the methodological obstacles important for domestic developments.

## Figures and Tables

**Table 1:** Peptide drugs developed in Russia and their clinical and pharmacological groups.

#	Original Russian Peptide Preparation	Clinical or Pharmacological Group
1	Dalargin (tyrosyl-d-alanyl-glycyl-phenylalanyl-leucyl-arginine)	Antiulcer drug with antisecretory activity
2	Thymogen (Glu–Try)	Immunostimulatory drug
3	Semax (Met–Glu–His–Phe–Pro–Gly–Pro)	Nootropic drug
4	Licopid (glucosaminylmuramil dipeptide)	Immunomodulator
5	Immunofan (arginyl-alpha-aspartyl-lysyl-valyl-tyrosyl-arginine)	Immunomodulator
6	Thymodepressin (dipeptide disodium salt; γ-d-glutamyl-d-tryptophan)	Immunosuppressive drug
7	Gepon (Thr–Glu–Lys–Lys–Arg–Arg–Glu–Thr–Val–Glu–Arg–Glu–Lys–Glu	Antiviral agent
8	Sedatin (Arg–Tyr-d-Ala–Phe–Gly)	Stress protector (for veterinary use)
9	Bestim (thymogen analog; γ-d-glutamyl-l-tryptophan)	Immunomodulator
10	Noopept (ethyl ester of N–phenyl–acetyl–l–prolyl–glycine).	Nootropic drug
11	Deltaran (tryptophanyl-alanyl-glycyl-glycyl-aspartyl-alanyl-seryl-glycyl-glutamic acid)	Stress protector, treatment of alcohol addiction
12	Stemokin (Ile–Glu–Trp)	Hematopoietic stimulant
13	Selank (l-threonyl-l-lysyl-l-prolyl-l-arginyl-l-prolylglycyl-l-proline)	Anxiolytic
14	Allokin alpha (alloferon; His–Gly–Val–Ser–Gly–His–Gly–Glu–His–Gly–Val–His–Gly)	Immunomodulator

## Data Availability

Not applicable.

## Supplementary Materials

The following is available online at https://www.mdpi.com/article/10.3390/pharmaceutics14040716/s1, Table S1: Approved peptide pharmaceuticals.
