# Supplementary material for: Development of Peptide Biopharmaceuticals in Russia
Source: Pharmaceutics. 2022 Mar 27;14(4):716. doi: 10.3390/pharmaceutics14040716 (PMC9030433; doi:10.3390/pharmaceutics14040716)
Supplement: Supplementary file 1 [file pharmaceutics-14-00716-s001.zip › pharmaceutics-1544045-supplementary.pdf]

pharmaceutics

Supplementary Materials: Development of Peptide Biopharmaceuticals in Russia

Vladislav I. Deigin, Elena A. Poluektova, Allan G. Beniashvili, Sergey A. Kozin and Yuri M. Poluektov

Table S1. Approved peptide pharmaceuticals.

Compound Name	Current Status	First Approval	Approved Indication(s)	Primary Therapeutic Area	Approved RoA	Chemical Basis
Corticotropin	Approved	1952	Multiple inflammatory diseases; West syndrome	CNS	Subcutaneous	Native
Vasopressin	Approved	1962	Vasodilatory shock; bleeding esophageal varices	Urology	Intravenous	Native
Oxytocin	Approved	1962	Labor induction; postpartum hemorrhage; lactation	Infertility GYN	Intramuscular; intravenous; intranasal	Native
Eledoisin	Approved	1970s	Dry eye	Ophthalmology	Topical	Native
Somatostatin	Approved	1970s	Bleeding esophageal varices	Hepatology and Critical Care	Intravenous	Native
Felypressin	Approved	1970s	Dental anesthesia adjunct	Pain	Subcutaneous	Analog
Calcitonin (salmon)	Approved	1971	Hypercalcemia; Paget's disease; osteoporosis	Bones and connective tissues	Intranasal; subcutaneous	Native
Ornipressin	Approved	1971	Bleeding esophageal varices	Hematology	Intravenous	Analog
Desmopressin	Approved	1972	Diabetes insipidus; nocturia	Urology	Oral; intranasal	Analog
Terlipressin	Approved	1978	Bleeding esophageal varices	Hepatology and Critical Care	Intravenous	Analog
Ambamustine	Withdrawn	1980s	Non-Hodgkin's lymphomas	Oncology	Intravenous	Heterologous
Tetracosactide	Approved	1980	Multiple inflammatory diseases	Endocrinology	Subcutaneous	Native
Elcatonin	Approved	1981	Osteoporosis	Bones and connective tissues	Intramuscular	Analog
Saralasin	Withdrawn	1981	Renovascular hypertension	Cardiovascular	Intravenous	Analog
cargutocin	Withdrawn	1982	Labor induction	Infertility and OB-GYN	Unknown (injectable)	Analog
buserelin	Approved	1984	Prostate cancer; endometriosis; assisted reproduction	Urology	Intranasal; subcutaneous	Analog
leuprorelin	Approved	1984	Prostate cancer; endometriosis; uterine fibroids; precocious puberty	Urology	Subcutaneous; intramuscular	Analog
thymopentin	Approved	1985	Various autoimmune and	Allergy and	Subcutaneous;	Analog

			infectious diseases	Immunology	intramuscular	
enalapril	Approved	1985	Heart failure; hypertension	Cardiovascular	Oral	Analog
triptorelin	Approved	1986	Prostate cancer; endometriosis; breast cancer; precocious puberty	Urology	Intramuscular	Analog
Calcitonin (human)	Approved	1986	Hypercalcemia; Paget's disease; osteoporosis	Bones and connective tissues	Intramuscular; intranasal; subcutaneous	Native
Goserelin	Approved	1987	Breast cancer; endometriosis; prostate cancer	Urology	Subcutaneous	Analog
Lisinopril	Approved	1987	Acute myocardial infarction; heart failure; hypertension	Cardiovascular	Oral	Analog
Octreotide	Approved	1988	Acromegaly; neuroendocrine tumors	Endocrinology	Subcutaneous; intramuscular; intravenous	Analog
Romurtide	Withdrawn	1989	Blood count support in cancer patients	Oncology	Subcutaneous	Analog
Glucagon	Approved	1989	Hypoglycemia	Metabolic	Subcutaneous	Native
Gonadorelin	Approved	1989	Assisted reproduction; primary amenorrhea	Infertility and OB-GYN	Subcutaneous	Native
Nafarelin	Approved	1990	Precocious puberty; endometriosis	Infertility and OB-GYN	Intranasal	Analog
Histrelin	Approved	1991	Prostate cancer; precocious puberty	Infertility and OB-GYN	Subcutaneous; intramuscular	Analog
Deslorelin	Withdrawn	1992	Endometriosis; precocious puberty	Infertility and OB-GYN	Subcutaneous	Analog
Lanreotide	Approved	1994	Acromegaly; neuroendocrine tumors	Endocrinology	Subcutaneous	Analog
Carperitide	Approved	1995	Acute decompensated heart failure	Cardiovascular	Intravenous	Native
Glatiramer	Approved	1996	Multiple sclerosis	CNS	Subcutaneous	Heterologous
Eptifibatide	Approved	1998	Acute coronary syndrome managed medically or with percutaneous coronary intervention	Cardiovascular	Intravenous	Analog
Cetrorelix	Approved	1999	Assisted reproduction	Infertility and OB-GYN	Subcutaneous	Analog
Ganirelix	Approved	1999	Assisted reproduction	Infertility and OB-GYN	Subcutaneous	Analog

bivalirudin	Approved	1999	Acute coronary syndrome; heparin-induced thrombocytopenia; thrombosis	Hematology	Intravenous	Analog
P-15	Approved	1999	Bone grafting	Orthopedics	Implanted	Analog
Atosiban	Approved	2000	Preterm labor	Infertility and OB-GYN	Intravenous	Analog
Taltirelin	Approved	2000	Spinocerebellar degeneration	CNS	Oral	Analog
Aviptadil	Approved	2000	Erectile dysfunction	Urology	Intracavernous	Analog
Nesiritide	Approved	2001	Acute decompensated heart Failure	Cardiovascular	Intravenous	Native
Carbetocin	Approved	2001	Postpartum hemorrhage	Infertility and OB-GYN	Intravenous	Analog
Teriparatide	Approved	2002	Osteoporosis	Bones and connective tissues	Subcutaneous	Analog
Abarelix	Withdrawn	2003	Prostate cancer	Urology	Intramuscular	Analog
Enfuvirtide	Approved	2003	HIV infection	Antimicrobial; Antiviral	Subcutaneous	Analog
Ziconotide	Approved	2004	Pain	Pain	Intrathecal	Native
Pramlintide	Approved	2005	Type 1 and type 2 diabetes	Metabolic	Subcutaneous	Analog
Exenatide	Approved	2005	Type 2 diabetes	Metabolic	Subcutaneous	Native
Romiplostim	Approved	2008	Immune thrombocytopenic purpura	Hematology	Subcutaneous	Heterologous
Degarelix	Approved	2008	Prostate cancer	Urology	Subcutaneous	Analog
Icatibant	Approved	2008	Hereditary angioedema in C1-esterase-inhibitor deficiency	Cardiovascular	Subcutaneous	Analog
Mifamurtide	Approved	2009	Osteosarcoma	Oncology	Intravenous	Analog
Liraglutide	Approved	2009	Type 2 diabetes	Metabolic	Subcutaneous	Analog
Tesamorelin	Approved	2010	HIV lipodystrophy	Metabolic	Subcutaneous	Analog
Peginesatide	Withdrawn	2012	Anemia associated with chronic kidney disease	Hematology	Intravenous; subcutaneous	Heterologous
Lucinactant	Approved	2012	Neonatal respiratory distress syndrome	Respiratory	Inhaled	Heterologous
Linaclotide	Approved	2012	Constipation-dominant irritible bowel syndrome; chronic idiopathic constipation	Gastroenterology	Oral	Analog
Pasireotide	Approved	2012	Acromegaly; Cushing’s disease	Endocrinology	Subcutaneous	Analog
Teduglutide	Approved	2012	Short bowel syndrome	Gastroenterology	Subcutaneous	Analog
Carfilzomib	Approved	2012	Multiple myeloma	Oncology	Intravenous	Analog

Lixisenatide	Approved	2013	Type 2 diabetes	Metabolic	Subcutaneous	Analog
Afamelanotide	Approved	2014	Erythropoietic protoporphyria	Dermatology	Subcutaneous	Analog
Albiglutide	Approved	2014	Type 2 diabetes	Metabolic	Subcutaneous	Analog
Dulaglutide	Approved	2014	Type 2 diabetes	Metabolic	Subcutaneous	Analog
Etelcalcetide	Approved	2016	Secondary hyperparathyroidism	Endocrinology	Intravenous	Heterologous
Plecanatide	Approved	2017	Chronic idiopathic constipation	Gastroenterology	Oral	Analog
Plecanatide	Approved	2017	Osteoporosis	Bones and connective tissues	Subcutaneous	Analog

RoA, routes of administration.
